# Maternal Psychosocial Stress Is Associated with Reduced Diversity in the Early Infant Gut Microbiome

**DOI:** 10.3390/microorganisms11040975

**Published:** 2023-04-08

**Authors:** Christopher L. Dutton, Felicien Masanga Maisha, Edward B. Quinn, Katherine Liceth Morales, Julie M. Moore, Connie J. Mulligan

**Affiliations:** 1 Department of Anthropology, University of Florida, 1115 Turlington Hall, P.O. Box 117305, Gainesville, FL 32611-7305, USA; cldutton@gmail.com (C.L.D.); felicienmaisha@gmail.com (F.M.M.); edwardquinn@ufl.edu (E.B.Q.); katherinemorales@ufl.edu (K.L.M.); 2 Genetics Institute, University of Florida, 2033 Mowry Rd, P.O. Box 103610, Gainesville, FL 32610-3610, USA; 3 Department of Biology, University of Florida, 220 Bartram Hall, P.O. Box 118525, Gainesville, FL 32611-8525, USA; 4 HEAL Africa Hospital, Rue Lyn Lusi No. 111, Goma BP 319, Democratic Republic of the Congo; 5 Department of Infectious Diseases & Immunology, College of Veterinary Medicine, University of Florida, Room V3-111B, P.O. Box 110880, Gainesville, FL 32611-4111, USA; juliemoore@ufl.edu

**Keywords:** maternal psychosocial stress, early infant gut, microbial diversity and composition, *Lactobacillus gasseri*, *Bifidobacterium pseudocatenulatum*

## Abstract

The developing infant gut microbiome is highly sensitive to environmental exposures, enabling its evolution into an organ that supports the immune system, confers protection from infection, and facilitates optimal gut and central nervous system function. In this study, we focus on the impact of maternal psychosocial stress on the infant gut microbiome. Forty-seven mother–infant dyads were recruited at the HEAL Africa Hospital in Goma, Democratic Republic of Congo. Extensive medical, demographic, and psychosocial stress data were collected at birth, and infant stool samples were collected at six weeks, three months, and six months. A composite maternal psychosocial stress score was created, based on eight questionnaires to capture a diverse range of stress exposures. Full-length 16S rRNA gene sequences were generated. Infants of mothers with high composite stress scores showed lower levels of gut microbiome beta diversity at six weeks and three months, as well as higher levels of alpha diversity at six months compared to infants of low stress mothers. Longitudinal analyses showed that infants of high stress mothers had lower levels of health-promoting *Lactobacillus gasseri* and *Bifidobacterium pseudocatenulatum* at six weeks compared to infants of low stress mothers, but the differences largely disappeared by three to six months. Previous research has shown that *L. gasseri* can be used as a probiotic to reduce inflammation, stress, and fatigue, as well as to improve mental state, while *B. pseudocatenulatum* is important in modulating the gut–brain axis in early development and in preventing mood disorders. Our finding of reduced levels of these health-promoting bacteria in infants of high stress mothers suggests that the infant gut microbiome may help mediate the effect of maternal stress on infant health and development.

## 1. Introduction

Much is still unknown about the process by which the human gut microbiome is established. The period immediately after birth is critical for the successful establishment of a healthy gut microbiome, which is essential for developing a strong immune system, protecting from infection and disease, and for establishing proper gut and central nervous system functioning [1]. The infant gut microbiome is highly dynamic and characterized by rapid evolution, high strain turnover, and high interindividual variation [2,3,4]. During the first six months, the microbiome shows low taxonomic diversity that is related to the limited number of microbes that can process breastmilk [3]. The introduction of solid foods and greater dietary diversity represents the second stage in gut microbiome development and is accompanied by an increase in microbial diversity [5]. After 36 months, the microbiome becomes more stable and less sensitive to environmental exposures [3].

Due to the active development of the gut microbiome immediately after birth, microbial composition and diversity are highly sensitive to environmental exposures during the first six months of life. Researchers have identified a range of factors that impact the early infant gut microbiome, including mode of delivery [6], preterm birth [7], breastfeeding and diet [8], and infection [9]. However, knowledge gaps remain in our understanding of infant gut microbiome development and the mechanistic links connecting microbiome dysbiosis with disease. Researchers have called for longitudinal studies, more investigation of environmental factors, and better description of healthy gut microbiomes [10,11].

Recently, researchers have begun studying the effect of psychosocial stressors on the gut microbiome as a possible mechanism through which mental and physical health is impacted [12]. Working with adults, Valles-Colomer et al. [13] reported associations of the gut microbiome with depression and other quality-of-life indicators. From an intergenerational perspective, mother–child interactions are particularly important for child development, and maternal stress during pregnancy has been shown to have wide-ranging effects on offspring health and development [14,15,16]. Hechler et al. [17] identified an association between maternal general anxiety and the maternal gut microbiome, which they propose as a possible mechanism to mediate the impact of maternal psychosocial stress on infant development and health. A limited number of studies have investigated the impact of maternal prenatal psychosocial stress on the infant gut microbiome and have identified associations with maternal psychological distress [18], maternal precarity [19], and a prenatal cumulative stress measure [20], but they were only able to analyze the infant microbiome at the genus or family level.

In the current study, we present a novel longitudinal analysis of the impact of maternal psychosocial stress on the infant gut microbiome based in the Democratic Republic of Congo (DRC). We focus on the first six months of life, when the infant gut is rapidly evolving and highly sensitive to environmental exposures. We sequenced the entire 16S rRNA gene using a new high-throughput sequencing methodology with single-nucleotide resolution and a near-zero error rate [21], which allows improved estimation of abundance levels and classification to the species and sub-species level. Our study provides critical data from an under-represented part of the world that will expand our understanding of the global microbiome [22,23,24]. Furthermore, women living in the DRC have experienced a wide range of psychosocial stressors and, thus, represent a model population to study the effect of maternal stress on offspring gut microbiota [25]. We hypothesize that infants of high stress mothers will have lower levels of microbiome diversity overall and lower abundance of healthy bacteria, such as *Lactobacillus* and *Bifidobacterium,* specifically.

## 2. Materials and Methods

### 2.1. Participant Recruitment and Consenting

Our research group has studied the effect of maternal stress on infant health in the DRC for more than ten years, and we have documented epigenetic effects of maternal stress on both the maternal and infant epigenome [24,25]. In this study, we tested for effects of maternal stress on the infant microbiome. Pregnant women were recruited at HEAL Africa hospital, Goma, DRC from March to October 2020. Inclusion criteria were singleton, uncomplicated, and vaginal delivery; no apparent infection or underlying medical condition present as determined by medical history and physical examination; and willingness to bring the infant to the hospital for regularly scheduled follow-up visits and whenever the infant was sick was also considered. Fifty-two women were originally enrolled in the study, and five women dropped out after delivery because they moved away, resulting in a final study sample of 47 mothers and infants.

Participant recruitment began with the informed consent process. Upon arrival at HEAL Africa, mothers were asked by a staff midwife about their interest in participating in the study. Participants were given the option of remaining in the maternity ward or moving to a private room. Most participants elected to remain in the maternity ward because they felt more comfortable there. An explanation of the study (and all subsequent interviews) was given in Congolese Swahili. Mothers were asked if they had any questions about the study and were told that they could withdraw from the study at any time. This study was conducted in accordance with the Declaration of Helsinki, and the protocol was approved by the University of Florida (Project #IRB202001503) and local ethics committees at the Université Libre des Grands Lacs (ULPGL/Goma) and HEAL Africa Hospital. Informed consent was obtained from all mothers participating in the study. All research was performed in accordance with relevant guidelines and regulations for the US and the DRC.

### 2.2. Medical Histories and Maternal Stress Measures

Following informed consent, medical histories, semi-structured interviews, and surveys were collected from mothers within one day of delivery, and infant stool samples were collected at follow-up visits, occurring at six weeks, three months, and six months. Medical histories at birth and at follow-up visits were collected by two staff midwives. A second research team, which was trained in conducting interviews with victims of violence, administered detailed interviews and validated surveys. General demographic, life history, and household conditions data were collected, and specific data on maternal stress, trauma, and mental health were collected using the following eight instruments: Violence Trauma and Pregnancy Trauma questionnaires developed based on questions in our previous studies [25,26], General Trauma and Sexual Trauma subsections of the Early Trauma Inventory Self-Report Short Form (ETISR-SF) [27,28], Perceived Stress Scale [29], intrusion subscale of the Impact of Event Scale to measure PTSD [30], Edinburgh Postnatal Depression Scale [31,32], and 20 state anxiety questions from the State/Trait Anxiety Inventory [33].

Stress scores, using different questionnaires, are often found to be weakly correlated, suggesting that different measures are tapping into different aspects of stress [17,20]. Thus, we created a composite stress score, as has been used for other types of psychosocial data [34] in order to capture the greatest range of stress exposures experienced by the women in our study. Scores on the eight instruments described above were standardized to a maximum score of 1 each and summed to create a composite stress measure (Table S1). K-means unsupervised clustering was used to create two categories of mothers with low (*n* = 26) and high (*n* = 21) composite stress scores. Statistical analyses compared information between infants from mothers with high versus low composite stress scores.

### 2.3. Sample Collection and Processing

Fecal samples were collected from infants at regularly scheduled follow-up visits at six weeks, three months, and six months. Seven samples from visits in which infants were diagnosed with malaria were removed from analyses. Fecal samples were collected using the OMNIgene Gut OMR-200 collection kit (DNAGenotek, Ottawa ON, Canada). OMR-200 homogenizes the sample in the collection tube and immediately preserves the DNA at room temperature for up to 60 days. Samples were stored in a −20 °C freezer and then shipped on ice to the University of Florida, where they were stored at −80 °C.

DNA was extracted from the fecal samples with the QIAamp PowerFecal Pro DNA extraction kit (Qiagen, Germantown, MD, USA) by following the manufacturer’s suggested protocol. DNA quantity and quality were assessed with a Denovix DS-ll. DNA samples were aliquoted to two 96-well plates with randomized sample placement and shipped frozen to the University of Illinois Roy J. Carver Biotechnology Center for library preparation and DNA sequencing. In short, 16S rRNA gene amplicons were generated from the barcoded full-length 16S rRNA gene primers from PacBio and the 2× Roche KAPA HIFI Hot Start Ready Mix. Amplicons were converted to a library with the SMRTBell Express Template Express Kit 2.0. The library was then sequenced twice on two separate SMRT Cell 8M trays on a PacBio Sequel IIe using the circular consensus sequence mode. The first cell was run with a 12 h movie time, and the second cell was run with a 15 hs movie time. The first cell produced 641,283 reads, and the second cell produced 2,104,832 reads. The average number of reads was 21,794 per sample, summed across the two cells.

Two negative controls (water blanks) and two extraction blanks were included and sequenced on each cell. All four controls returned zero reads. Two extraction replicates (the same sample was extracted twice) and three sequence replicates (the same sample extract was used twice to generate two different libraries) were also sequenced on each cell. All replicate samples showed good agreement between their microbial community composition. Three mock community samples (D6306, Lot 195709, Zymo Research, Irvin, CA, USA) were also included and sequenced on each cell. The three mock communities showed good agreement at the genus level for their microbial community composition. However, several taxa were misidentified at the species level. *Bacillus subtilis* was misidentified as *Bacillus intestinalis,* and *Escherichia coli* was misidentified as two different *Shigella* species (S. *boydii* and *S. flexneri*). Five different sequences of the genus *Limnosilactobacillus* were unidentified. We performed BLAST searches for each of the sequences misidentified against the NCBI nucleotide database on 3 November 2022 and confirmed the misidentification of those sequences. Callahan et al. has reported similar misidentifications with this same mock community and workflow for *B. subtilis* and *L. fermentum* [21]. *E. coli* and the two *Shigella* species are closely related and are traditionally difficult to identify at the species level [35].

### 2.4. Statistical Analyses

The statistical platform R (v. 4.1.3) was used for all data processing and statistical tests [36]. The DADA2 v. 1.24.0 R software package was used to resolve exact amplicon sequence variants (ASV) from the long amplicon reads using the published workflow for PacBio full 16S rRNA gene sequencing [21]. The error rate was learned for each separate sequence cell run, and then the reads from both cells were merged after error learning to infer the real biological sequence variants. Taxonomy was assigned down to the species level using the SILVA v128 database with the naïve Bayesian classifier [37,38].

The phyloseq and microeco packages were used to process the data [39,40]. To compare the categorized groups of infants of high and low stress mothers, a Kruskal Wallis rank sum test was used on maternal age, BMI, height, weight and infant sex, length, and weight. Microbial alpha diversity was calculated using Shannon’s richness, and beta diversity was calculated using the Bray-Curtis distance metric at each age. Shannon’s richness and Bray-Curtis distance were used to compare each age to one another using a Kruskal-Wallis rank sum test, followed by Dunn’s test for multiple pairwise comparisons where appropriate. A principal coordinates analysis (PCoA) was calculated on the Bray-Curtis distance for each age and ordinated using the first two axes. At each age, Shannon’s richness and Bray-Curtis distances were compared using the Kruskal-Wallis rank sum test between infants of high and low stress mothers. Correlation coefficients were calculated between covariates and taxa. Any covariate with minimal variation was removed from subsequent analyses. Infant sex and infant antibiotic use showed significant differences in bacterial beta diversity and were included as covariates in subsequent analyses.

Differentially abundant species between infants of mothers with low versus high stress scores were determined at each age while controlling for infant sex and antibiotic use using the ANCOM-BC v. 1.4.0 software package in R [41]. ANCOM-BC has been shown to accurately estimate sampling fractions, provide individual *p*-values and confidence intervals, and works well with samples greater than ten within a linear regression framework [41,42]. The Holm-Bonferroni method [43] was used to calculate the false discovery rate (FDR). The ComplexHeatmap package [44] was used to generate a clustered heatmap for each age.

For species that were differentially abundant in the infant gut at two or three ages, longitudinal patterns across ages were examined between infants of mothers with high versus low composite stress scores using the SplinectomeR v. 0.1.0 software package [45]. SplinectomeR was developed for longitudinal microbiome data and uses weighted local polynomials to model species abundance data across time and to test whether two categories of individuals follow a more different trajectory over time than would be expected by random chance. This approach is robust to partially missing data yet maintains individual observations [45]. No covariates were included (SplinectomeR does not allow covariates as it generates probability distributions), and analyses were run for 999 permutations.

## 3. Results

### 3.1. Sample Characteristics

Data and samples sufficient for analysis were collected from 47 babies at six weeks, three months, and six months. Average maternal age was 27.3 years, and 44.7% of infants were female. Average maternal BMI was 27.9, and average infant birthweight was 3200 gm (Table 1). Maternal composite stress scores ranged from 0.91 to 5.02 (Table S1). Twenty-six mothers were in the low stress category (0.91–3.18 composite stress score), and 21 were in the high stress category (3.32–5.02 composite stress score). None of the covariates were significantly different between high and low composite stress groups or were significantly correlated with the maternal composite stress score. Many covariates showed little variation and were not included in analyses, e.g., all babies were being breast-fed at six months, only two babies were eating solid foods at six months, and only two mothers smoked during their pregnancy (Table 1 and Table S2).

### 3.2. Infant Gut Microbial Relative Abundance and Diversity

**Figure 1:**
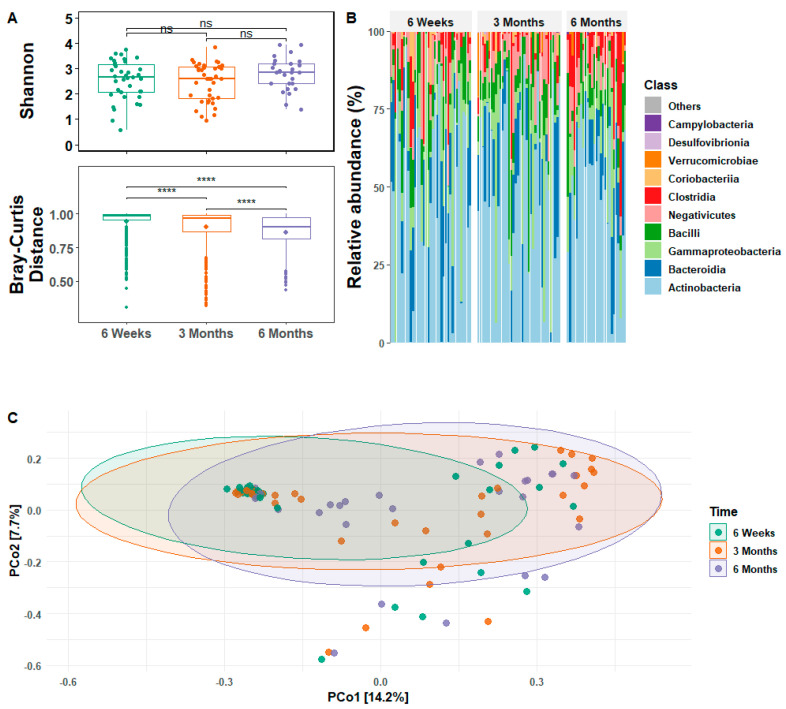
Relative abundance and diversity of infant gut microbiota across three ages. (**A**). Microbial alpha diversity, calculated as Shannon’s richness, is plotted in the top row, and beta diversity, calculated as Bray-Curtis Distance, is plotted in the bottom row. Both measures are indicated for all three ages. **** indicates *p* < 0.0001, ‘ns’ indicates no significance. (**B**). Relative abundance (%) is shown on the left, and each individual is depicted as a vertical bar grouped by age. The top 10 microbial classes are coded by color, with the least abundant class indicated at the top of each bar. Remaining classes are grouped together and labeled as “others” in gray. (**C**). Principal coordinate analysis (PCoA) of Bray-Curtis distances between the three ages. Circles represent 95% confidence ellipses. The relative sizes of the x and y axes are normalized, so they represent the same amount of variation for the first two PC axes to more faithfully represent the distances between samples, i.e., the x axis is roughly twice the length of the y axis.

Analysis of the relative abundance and diversity of gut microbiota revealed the dynamic nature of the infant gut microbiome over the first six months of life (Figure 1). Overall, microbial alpha diversity (Shannon’s Richness, or within group diversity) did not differ significantly as a function of infant age, but beta diversity (Bray-Curtis Distance, or between group diversity) differed significantly between infants at all three ages and declined over time (Figure 1A). *Actinobacteria* were most abundant, with an average relative abundance of 40 to 50% across all ages (Figure 1B). *Bacteroidia* and *Gammaproteobacteria* were the next most abundant, present at ~20% each at six weeks and declining slightly at three and six months. *Bacilli* ranged from 10 to 15% across ages. *Negativicutes* was present at 5 to 10% across all ages. *Clostridia*, of which ~25% of species are pathogenic, increased to a high of >10% at six months. *Coriobacteria*, *Verrucomicrobiae*, *Campylobacteria*, and *Desulfovibrionia* were present at very low levels and were absent in most individuals. The first two components of a PCoA captured 21.9% of the variation in Bray-Curtis distances and illustrated the changes in between group diversity as the infant gut developed during the first six months of life (Figure 1C).

### 3.3. Infant Gut Microbial Diversity and Maternal Stress

**Figure 2:**
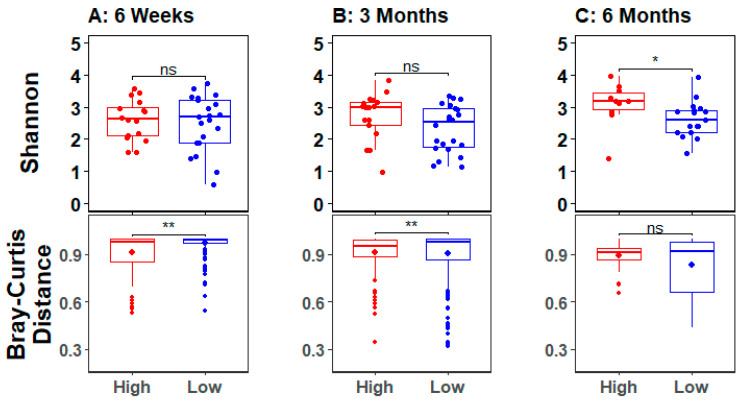
Gut microbial beta and alpha diversity at three ages for infants of high and low stress mothers. Microbial alpha diversity, calculated as Shannon’s richness, is plotted in the top row, and beta diversity, calculated as the Bray-Curtis Distance, is plotted in the bottom row for infants of high and low stress mothers at six weeks (**A**), three months (**B**), and six months (**C**). In the Shannon plots, points indicate individual samples, and, in the Bray-Curtis plots, the central diamond indicates the mean and points outside of the quartiles represent outliers, i.e., there are fewer outliers at six months compared to the other ages. * indicates *p* < 0.05, ** indicates *p* < 0.01, and ‘ns’ indicates no significance.

In order to analyze infant gut microbial diversity relative to maternal stress, alpha and beta diversity measures were calculated for infants of mothers dichotomized by high and low composite stress scores. At six weeks and three months, beta diversity was significantly influenced by maternal stress (Figure 2). Specifically, infants of mothers with high stress had lower between group diversity relative to infants of low stress mothers. At six months, there was no difference in beta diversity, but alpha diversity was significantly reduced in infants of low versus high stress mothers.

**Figure 3:**
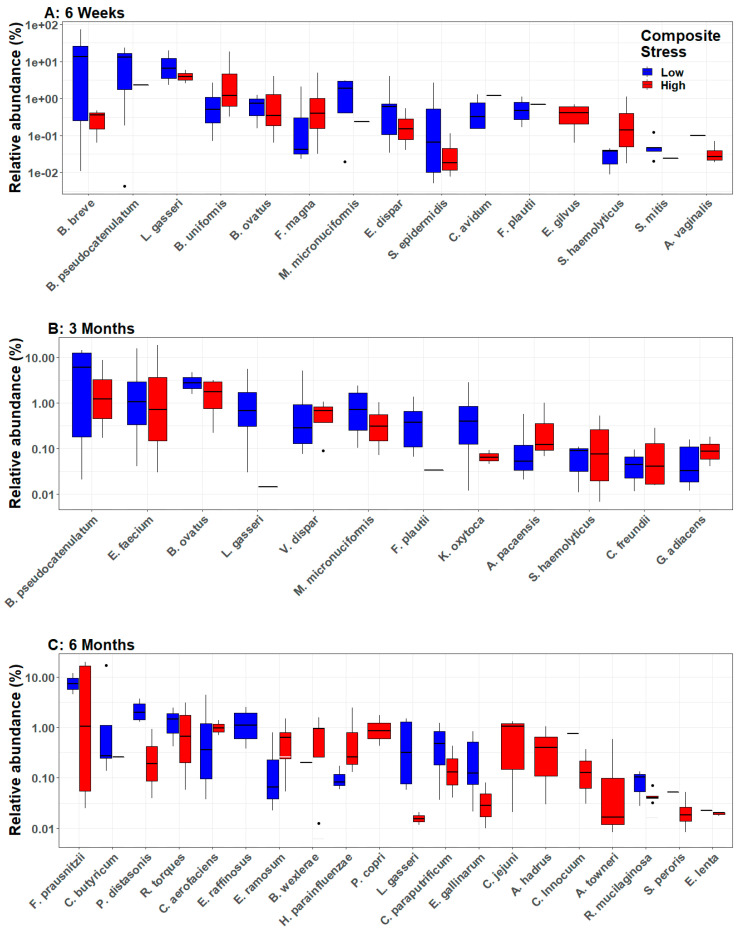
Differential abundance analysis of gut microbiota of infants born to high and low stress mothers. Box plots depict the relative abundance (%) of all bacteria that were significantly differentially abundant, shown for groups of infants of high and low stress mothers. The plots depict % relative abundance on a log scale (individuals with zero relative abundance of a particular taxa are not shown since log (0) is undefined). Analyses controlled for infant sex and antibiotic use, and compared infants of high and low stress mothers at (**A**) six weeks, (**B**) three months, and (**C**) six months.

Differential abundance analyses of the infant gut microbiome revealed multiple bacterial species that showed significantly different abundances between infants born to high versus low stress mothers (Figure 3 and Figure S1). Fifteen species were differentially abundant between infants of high and low stress mothers at six weeks, 12 species were differentially abundant at three months, and 20 species were differentially abundant at six months. *Lactobacillus gasseri* was the only species that was differentially abundant at all three ages and showed significantly lower abundance in infants of high stress mothers compared to low stress mothers. *Bifidobacterium pseudocatenulatum* also showed lower abundance in infants of high stress mothers compared to low stress mothers, but only at six weeks and three months. *Veillonella dispar, Bacteroides ovatus, Megasphaera micronuciformis*, and *Flavonifractor plautii* were the only other species that showed significantly different abundance at more than one age, specifically at six weeks and three months. For these four species, the abundance differences between infants of high versus low stress mothers were smaller, and for *V. dispar* and *F. plautii,* the relative abundance switched when comparing infants of high versus low stress mothers at six weeks and three months.

### 3.4. Longitudinal Analyses

Differential abundance analyses identified significant differences in bacterial abundance between infants of high and low stress mothers. Longitudinal analyses were conducted to test the extent to which differences in abundance persisted over time. Thus, longitudinal analyses were conducted on all species that showed significant differential abundance between infants of high and low stress mothers at two or three ages.

**Figure 4:**
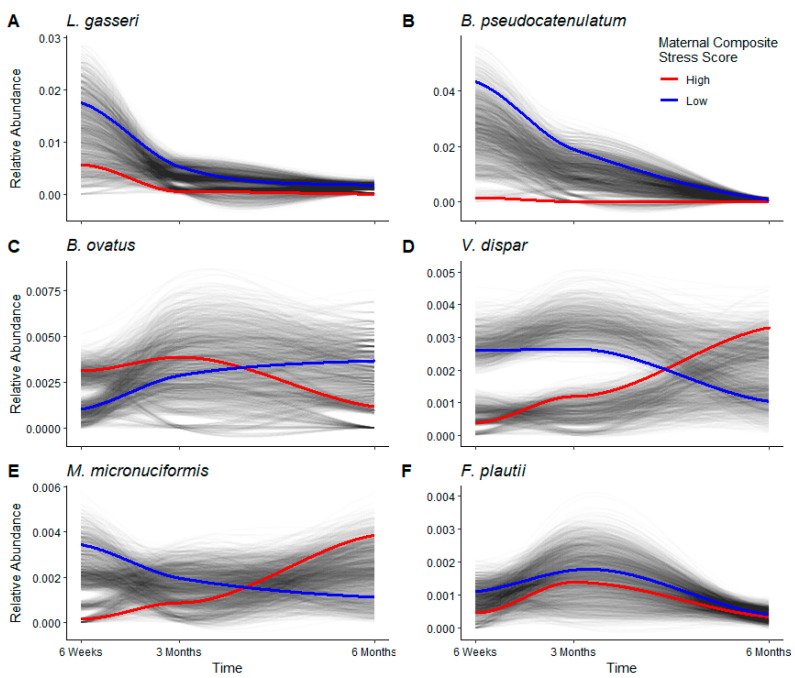
Longitudinal analysis of changes in relative abundance of bacterial species. Relative abundance is plotted on the Y axis, and time is plotted on the X axis. Infants of mothers with high stress (red line) and low stress (blue line) were compared for relative abundance of (**A**) *L. gasseri*, (**B**) *B. pseudocatenulatum*, (**C**) *B. ovatus,* (**D**) *V. dispar*, (**E**) *M. micronuciformis*, and (**F**) *F. plautii*. Note that the scale for relative abundance on the Y axis is an order of magnitude larger in **A** and **B** relative to **C**–**F**.

As reported above, *L. gasseri* was the only bacterium to show large and significant differential abundance between infants of high and low stress mothers at all three ages (Figure 3). When relative abundance was analyzed longitudinally, *L. gasseri* showed a large variance in all infants and a large difference in relative abundance between babies of high and low stress mothers at six weeks, but the variance and difference began to tighten around three months (Figure 4A). Across all three ages, infants of high stress mothers had a lower relative abundance of *L. gasseri* compared to infants of low stress mothers. Individual infants showed great variation at six weeks compared to three and six months, suggesting the infant gut microbiome was still evolving with respect to *L. gasseri* relative abundance during the first three months, in contrast to the relative stability seen between three and six months.

As reported above, in differential abundance analyses, *B. pseudocatenulatum* showed increased abundance in infants of low stress mothers at six weeks and three months (Figure 3). In longitudinal analyses, this species showed a large variance in all infants and a large difference in relative abundance between babies of high and low stress mothers at six weeks, but the variance and difference were greatly reduced by six months (Figure 4B). Infants of high stress mothers showed low and unchanging relative abundance of *B. pseudocatenulatum* at all three ages.

The other four species that showed small, but significant, differential abundance at six weeks and three months (Figure 3A,B; *V. dispar*, *B. ovatus*, *M. micronuciformis*, *F. plautii*) showed consistently low abundance and high variance across the first six months in longitudinal analyses (Figure 4C–F). *B. ovatus* showed higher abundance in infants of high stress mothers at six weeks, but it switched to higher abundance in infants of low stress mothers after three months (Figure 4C). The other three species are pathogenic, and two species switched in relative abundance with respect to maternal stress, i.e., *V. dispar* and *M. micronuciformis* were more abundant in infants of low stress mothers at six weeks, but more abundant in infants of high stress mothers after three months (Figure 4D,E). The switching abundance of these species relative to maternal stress is reflected in both the differential abundance and longitudinal analyses and suggests possible dysbiosis in the infant gut microbiome in response to maternal stress.

## 4. Discussion

Using a longitudinal study design, we report that maternal psychosocial stress is associated with decreased abundance of health-promoting bacteria, such as *L. gasseri* and *B. pseudocatenulatum*. Furthermore, dysbiosis of pathogenic bacteria, such as *V. dispar* and *M. micronuciformis,* was observed during the first six months in the developing infant gut microbiome. Thus, our results confirm our hypothesis and demonstrate the effect of maternal stress on the next generation through the infant gut microbiome. These results support the thesis that the infant gut microbiome plays a role in mediating the impact of maternal stress on infant health and development. An unhealthy gut microbiome may play a direct causal role in disease risk and/or may make an individual more susceptible to the many stressors that cause disease [10].

Due to the active development of the infant gut microbiome immediately after birth, microbial composition and diversity are highly variable and sensitive to environmental exposures during the first six months of life. Our results, from an understudied part of the world, demonstrate the universality of the dynamic nature of the early infant gut microbiome. In the current study, microbial beta diversity was significantly different between infants at all three ages (Figure 1A). When analyzed with respect to maternal stress, infants of high stress mothers showed significantly reduced beta diversity at six weeks and three months, as well as significantly increased alpha diversity at six months (Figure 2). Differential abundance analyses showed a transition between three and six months such that the number of significant differentially abundant bacteria almost doubled during this time period (Figure 3). Of the 20 differentially abundant species at six months, longitudinal analyses revealed that 14 were elevated in infants of high stress mothers (see SplinectomeR.html file at https://figshare.com/s/a11994ad0dcc1524a60a (accessed on 4 April 2023)), and at least eight show some pathogenicity [46,47,48,49,50,51,52], suggesting the increased alpha diversity in infants of high stress mothers at six months may reflect increased levels of pathogenic bacteria. Furthermore, relative abundance heat maps showed increased clustering at six months of infant gut bacteria by maternal stress, i.e., increased clustering of infants of high versus low stress mothers (Figure S1), an indication of reduced interindividual variation that is consistent with the lack of significant difference in beta diversity detected at six months. Introduction of solid foods around six months is thought to drive the diversification of the infant gut microbiome [5,8]. However, only two infants in our study were consuming solid foods at six months. Yet, we saw a transition in the gut microbiome, suggesting that, in our study, population diet alone does not drive the diversification of the infant gut microbiome at six months.

A significant reduction in healthy bacteria, *L. gasseri* and *B. pseudocatenulatum,* in the gut of infants of high stress mothers (Figure 3 and Figure 4), may increase the infants’ susceptibility to disease. Three previous studies have reported associations between maternal stress and the infant gut microbiome, but those studies used older sequencing technologies that could only classify infant gut bacteria to the genus level [18,19,20]. Older short-read sequencing technologies had high error rates and often led to misclassification at the species level and over-estimation of taxa number and diversity levels [53,54]. Using new high-throughput amplicon sequencing [21], we were able to generate high-fidelity species-level microbiome data that allowed improved characterization of the infant gut microbiome. Thus, we specify the importance of *L. gasseri* and *B. pseudocatenulatum* with respect to maternal stress in contrast to the other 43 and 79 species in the *Lactobacillis* and *Bifidobacterium* genera, respectively.

Evidence for the importance of *L. gasseri* and *B. pseudocatenulatum* in health is reflected in emerging interventions. Building on the evidence for an altered microbiome in individuals with mental health disorders and the patchy performance of medications to treat conditions such as depression and anxiety, researchers are developing probiotics and fecal transplants to seed guts with healthy bacteria [55]. In a placebo-controlled clinical trial, athletes who took daily heat-inactivated *L. gasseri* showed reduced fatigue and improved mental state, as well as increased alpha and beta diversity of fecal microbiota [56]. Medical students who took daily paraprobiotic *L. gasseri* experienced improved sleep, reduced stress, lowered salivary cortisol, and suppression of stress-responsive microRNAs [57]. *B. pseudocatenulatum* may play an important role in modulating the gut–brain axis in early development and ameliorating the effects of mood disorders. Mice fed a diet including *B. pseudocatenulatum* showed reduced anhedonia and reduced stress response to physical and social stress in an obesity model [58], as well as lower anxiety, reduced intestinal inflammation, and reversed intestinal dysbiosis, with long-lasting effects into adulthood in a model of early chronic stress [59]. Both *L. gasseri* and *B. pseudocatenulatum* also appear to play a role in resistance from infectious disease, as mouse studies reported that fecal transplants from malaria-resistant mice conferred resistance to malaria concurrently with increased abundance of *Lactobacillis* and *Bifidobacterium* [60,61].

Four other bacteria showed small, but significant, changes in relative abundance in association with maternal stress. *B. ovatus* has been suggested to play an important role in regulating the immune system and intestinal inflammation [62,63]. The switching abundance of *B. ovatus* in relation to maternal stress and the lower abundance in infants of high stress mothers beginning after three months may indicate dysbiosis and suboptimal development of the gut, immune, and central nervous systems. The other three species have not been well studied, but they are thought to be at least slightly pathogenic. *Veillonella* sp. has been associated with many infections, and species-level identification has recently enabled the association of *V. dispar* with prosthetic joint infections [64]. *M micronuciformis* is not well studied [65], but there is one clinical report of isolation from a human liver abscess [66]. *F. plautii* is not well studied clinically, but two cases of *F. plautii* infection in immunosuppressed patients have been described [67,68]. Future studies with species-level microbiome sequence data will help elucidate the role of these bacteria in establishing the infant gut microbiome.

The strengths of our study include a longitudinal study design over the first six months of life and generation of high-fidelity, species-level sequence data from the complete 16S rRNA gene. In addition, we provide critical data from an under-represented part of the world to expand our understanding of the global microbiome. Our study was limited by a small sample size of 47 mother-infant dyads, although our longitudinal study design with samples from three follow-up visits improved the sample size and allowed us to better control for noise when plotting trajectories. Additionally, as with all human studies, our results are correlative, not causative. However, our results provide specific recommendations to test for causative effects of psychosocial stress on *L. gasseri* and *B. pseudocatenulatum* abundance and related health outcomes in a murine model. Development of the infant gut microbiome is complex and impacted by many factors, in addition to maternal psychosocial stress; our study accounted for the variables known to be important (mode of delivery, breastfeeding and solid food, infant sex, antibiotic use, and infection), but it is possible that there are additional variables that should be included. The high levels of *Bifidobacterium and Lactobacillus,* seen across all ages, are consistent with vaginal delivery and breastfeeding [3,4,69], which were inclusion criteria for our study, suggesting that we have included the most important variables. Expansion of the current work to include assessment of fecal microbiota at timepoints later in infancy, when introduction to solid foods and exposure to other environmental factors will increase, will be important to assess the durability of the impact of maternal stress on the infant gut microbiome. Finally, it will be critical for future studies in this population to examine the maternal gut and/or vaginal microbiota, which are critical in seeding the infant gut microbiome [70], as well as the extent to which these microbiomes are modified by maternal indicators of stress.

## 5. Conclusions

We provide the first species-level evidence that maternal psychosocial stress is associated with decreased abundance of health-promoting bacteria, such as *L. gasseri* and *B. pseudocatenulatum,* as well as the dysbiosis of pathogenic bacteria, such as *V. dispar* and *M. micronuciformis,* in the developing infant gut microbiome. The strengths of our study include a longitudinal research design and generation of high-fidelity, species-level sequence data for the full 16S rRNA gene that allowed improved characterization of the infant gut microbiome. We also provide critical microbiome composition and diversity data from an understudied part of the world [71,72]. Our results suggest that the infant gut microbiome may play a role in mediating the impact of maternal stress on infant health and development.

## Figures and Tables

**Table 1:** Study population characteristics.

Variable	High Composite Stress	Low Composite Stress	Total	*p*-Value *	Total
	(N = 21)	(N = 26)	(N = 47)		(N = 47)
**Mother**					
**Age (years)**					
Mean (SD)	26.71 (8.07)	27.85 (5.99)	27.34 (7.02)	0.55	27.34 (7.02)
**BMI**					
Mean (SD)	27.15 (3.33)	28.58 (4.27)	27.93 (3.93)	0.35	27.93 (3.93)
**Height (cm)**					
Mean (SD)	159 (7.5)	159 (5.1)	159 (7.5)	0.34	159 (7.5)
**Weight (kg)**					
Mean (SD)	68.7 (9.5)	72.3 (13.0)	70.7 (11.7)	0.43	70.7 (11.7)
**Alcohol in pregnancy**					
No	15	18	33		33
Yes	6	8	14		14
**Smoking in pregnancy**					
No	20	25	45		45
Yes	1	1	2		2
**Infant**					
**Sex**					
Female	9	12	21	0.82	21
Male	12	14	26		26
**Length (cm)**					
Mean (SD)	46.9 (1.3)	47.3 (1.5)	47.1 (1.4)	0.45	70.7 (11.7)
**Weight (g)**					
Mean (SD)	3142 (389)	3263 (343)	3209 (369)	0.22	3209 (369)
**Breastfed**					
No	0	0	0		0
Yes	21	26	47		47
**Solid foods by 6 months**					
No	20	25	45		45
Yes	1	1	2		2
**Antibiotic usage by 6 months**					
No	16	22	38		38
Yes	5	4	9		9

* *p*-values are shown for variables that showed variation and were included in any analyses.

## Data Availability

The microbial DNA sequences supporting the conclusions of this study are available in the NCBI repository as BioProject ID PRJNA903474, https://www.ncbi.nlm.nih.gov/bioproject/?term=PRJNA903474. The R markdown files, HTML files, input metadata files, and microbial sequences plus associated metadata supporting the conclusions of this study are available in the figshare repository, https://figshare.com/s/a11994ad0dcc1524a60a.

## Supplementary Materials

The following supporting information can be downloaded at: https://www.mdpi.com/article/10.3390/microorganisms11040975/s1, Table S1: Scores from eight measures of stress and trauma and composite stress score for each mother; Table S2: Maternal and infant characteristics per dyad; Figure S1: Differential abundance analysis of gut microbiota of infants born to high and low stress mothers. Heat maps indicate the relative abundance (%) of all bacteria that were significantly differentially abundant between infants of high and low stress mothers; bacterial taxa (clustered on the left and labeled on the right) and individual infants (clustered on the top and labeled on the bottom) are clustered based on similar microbial abundance levels. Colors indicate differences in relative abundance, microbial phyla, and high/low maternal composite stress. Analyses controlled for infant sex and antibiotic use, and compared infants of high and low stress mothers at (A) six weeks, (B) three months, and (C) six months.
