# Supplementary figures and images for: Maternal Psychosocial Stress Is Associated with Reduced Diversity in the Early Infant Gut Microbiome
Source: Microorganisms. 2023 Apr 8;11(4):975. doi: 10.3390/microorganisms11040975 (PMC10142543; doi:10.3390/microorganisms11040975)

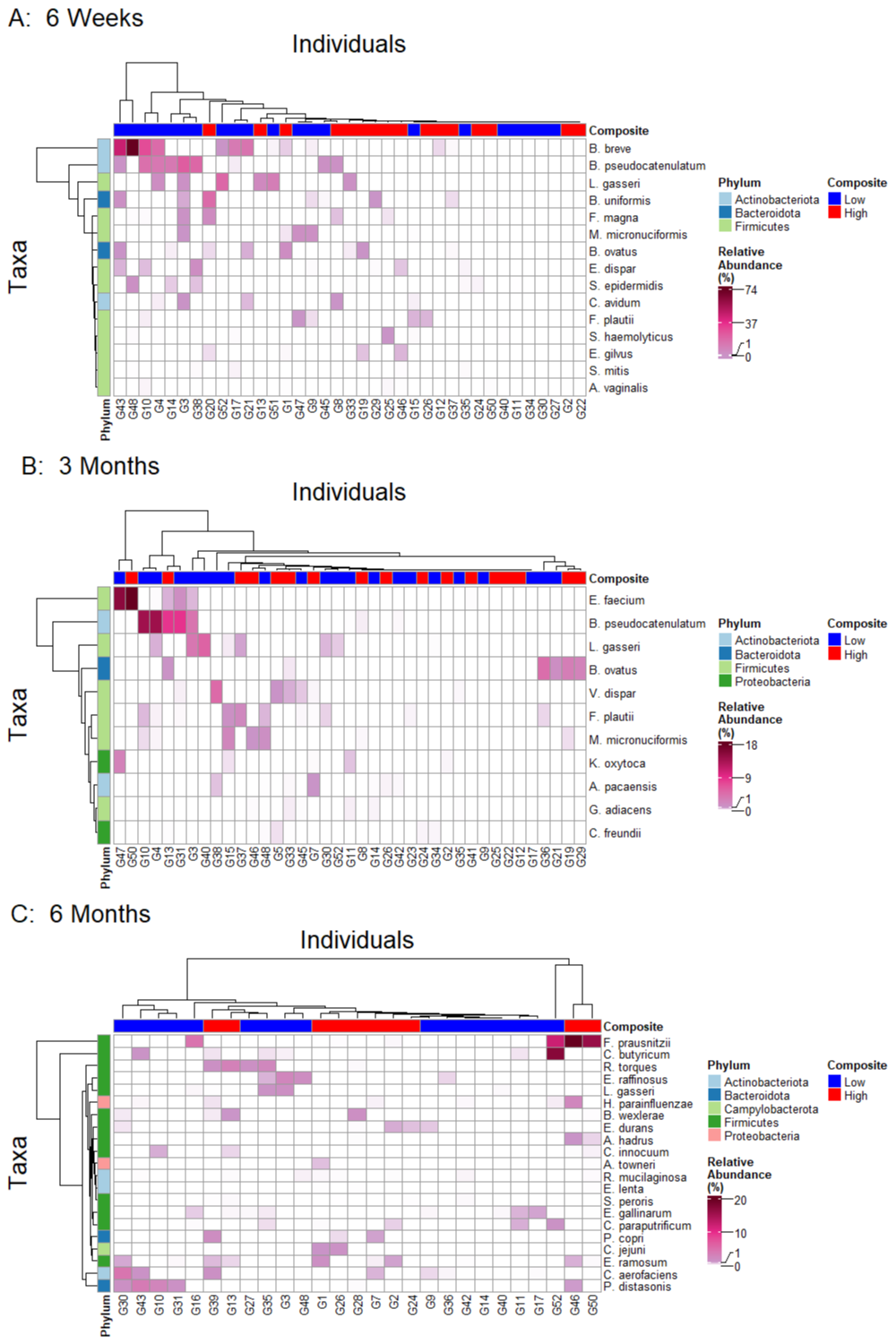

Supplement: Supplementary file 1 [file microorganisms-11-00975-s001.zip › Figure S1.png]
